# Supplementary material for: No Evidence for Myocarditis or Other Organ Affection by Induction of an Immune Response against Critical SARS-CoV-2 Protein Epitopes in a Mouse Model Susceptible for Autoimmunity
Source: Int J Mol Sci. 2023 Jun 8;24(12):9873. doi: 10.3390/ijms24129873 (PMC10298153; doi:10.3390/ijms24129873)
Supplement: Supplementary file 1 [file ijms-24-09873-s001.zip › ijms-2425796-supplementary.pdf]

Supplementary Material

Supplementary Table

Supplementary Figures

1 Supplementary Figures and Tables

1.1 Supplementary Tables

Table S1: Based on the B-cell epitope prediction [70], 38 generated Coronavirus peptide sequences were synthesized, grouped in 8 clusters and compared to the whole SARS-COV-2 spike protein sequence (https://www.ncbi.nlm.nih.gov/protein/BCA87361.1). Single and pooled SARS-CoV-2 peptide sequences were administered to A/JOla mice (length of the peptides listed as amino acid number). The corresponding sample size of used animals is indicated after the sequence as n. Colored sequences indicate location of the peptides in the whole SARS-CoV-2 protein. The underlined sequence parts show the sequences used for synthesis and immunization.

Peptide number and protein sequence			Length	Sequence Pool
1	VLLPLVSSQCVNLTTRTQLP	n=6	20	SPG1 (n=6)
2	SNVTWFHAIHVSGTNGTKRF	n=6	20	
3	TTLDSKTQSLLIVNNATNVV	n=6	20	
4	HKNNKSWMESEFRVYSSANNCTF	n=6	23	
5	SYLTPGDSSSGWTAGAAAYY	n=6	20	
6	TFLLKYNENGTITDAVDCAL	n=6	20	
7	FTVEKGIYQTSNFRVQPTES	n=6	20	SPG2 (n=6)
8	KRISNCVADYSVLYNSASFST	n=6	21	
9	FTGCVIAWNSNNLDSKVGGN	n=6	20	
10	RDISTEIYQAGSTPCNGVEG	n=6	20	
11	GPKKSTNLVKNKCVNFNFNGLTGTGVLTESN	n=6	31	
12	FGGVSVITPGTNTSNQVAVLYQDVNCTEV	n=7	29	
13	PTWRVYSTGSNVFQTRAGCL	n=7	20	
14	AGCLIGAEHVNNSYECDIPI	n=6	20	SPG3 (n=6)
15	SYQTQTNSPRRARSVASQSIIAYTMSLGAENSVAYSNNSIA	n=6	41	
16	TISVTTEILPVSMTKTSVDCT	n=6	21	
17	CTMYICGDSTECSNLLLQYG	n=6	20	
18	LLQYGSFCTQLNRALTGIAV	n=6	20	SPG4 (n=6)
19	ALTGIAVEQDKNTQEVFAQV	n=6	20	
20	LLTDEMIAQYTSALLAGTIT	n=6	20	
21	VLYENQKLIANQFNSAIGKIQDSLSSTASALG	n=6	32	
22	LQDVVNQNAQALNTLVKQLSSNFGAISS	n=6	28	
23	RLITGRLQSLQTYVTQQLIR	n=6	20	
24	IRASANLAATKMSECVLGQS	n=6	20	SPG5 (n=6)
25	PQIITTDNTFVSGNCDVVIG	n=6	20	
26	ELDKYFKNHTSPDVDLGDIS	n=6	20	
27	LGDISGINASVVNIQKEIDR	n=6	20	
28	IDRLNEVAKNLNESLIDLQE	n=6	20	
29	LIAIVMVTIMLCCMTSCCSC	n=6	20	
30	FGGPSDSTGSNQNGERSGAR	n=6	20	

Peptide number and protein sequence			Length	Sequence Pool
31	RGGSQASSRSSSRSRNSSRNSTPGSSRGTSPARMAGNGG	n=6	39	NPG (n=6)
32	SKMSGKGQQQQGQTVTKKSA	n=6	20	
33	LDDFSKQLQQSMSSADSTQA	n=6	20	
34	RLFARTRSMWSFNPETNILL	n=6	20	MPG (n=6)
35	ITVATSRTLSYYKLGASQRVAGDSGFAA	n=6	28	
36	IGNYKLNTDHSSSSDNIAL	n=6	19	
37	MYSFVSEETGTLIVNSVLLFLAFVVFLLVTLAILTALRLCA	n=6	41	EPG (n=6)
38	TALRLCAYCCNIVNVSLVKPSFYVYSRVKNLNSSRVPDLLV	n=6	41	

SARS-CoV-2 Protein Sequence	Structure
MFVFLVLLPLVSSQCVNLTTRTQLPPAYTNSFTRGVYYPDKVFRSSVLYSTQDLFLPFFS<br>NVTWFHAIHVSGTNGTKRFDNPVLPFNDGVYFASTEKSNIIRGWIFGTTLDSKTQSLLI<br>VNNATNVVIKVCEFQFCNDPFLGVYYHKNNKSWMESEFRVYSSANNCTFEYVSQPFL<br>MDLEGKQGNFKNLREFVFKNIDGYFKIYSKHTPINLVRDLPQGFSALEPLVDLPIGINIT<br>RFQTLLALHRSYLTPGDSSSGWTAGAAAYYVGYLQPRTFLLKYNENGTITDAVDCAL<br>DPLSETKCTLKSFTVEKGIYQTSNFRVQPTESIVRFPNITNLCPFGEVFNATRFASVYAW<br>NRKRISNCVADYSVLYNSASFSTFKCYGVSPTKLNDLCFTNVYADSFVIRGDEVRQIAP<br>GQTGKIADYNYKLPDDFTGCVIAWNSNNLDSKVGGNYNYLYRLFRKSNLKPFERDIS<br>TEIYQAGSTPCNGVEGFNCYFPLQSYGFQPTNGVGYQPYRVVVLSFELLHAPATVCGP<br>KKSTNLVKNKCVNFNFNGLTGTGVLTESNKKFLPFQQFGRDIADTTDAVRDPQTLEIL<br>DITPCSFGGVSVITPGTNTSNQVAVLYQDVNCTEVPVAIHADQLTPTWRVYSTGSNVF<br>QTRAGCLIGAEHVNNSYECDIPIGAGICASYQTQTNSPRRARSVASQSIIAYTMSLGAE<br>NSVAYSNNSIAIPTNFTISVTTEILPVSMTKTSVDCTMYICGDSTECSNLLLQYGSFCTQL<br>NRALTGIAVEQDKNTQEVFAQVKQIYKTPPIKDFGGFNFSQILPDPSKPSKRSFIEDLLF<br>NKVTLADAGFIKQYGDCLGDIAARDLICAQKFNGLTVLPPLLTDEMIAQYTSALLAGT<br>ITSGWTFGAGAALQIPFAMQMAYRFNGIGVTQNVLYENQKLIANQFNSAIGKIQDSLS<br>STASALGKLQDVVNQNAQALNTLVKQLSSNFGAISSVLNDILSRLDKVEAEVQIDRLI<br>TGRLQSLQTYVTQQLIRAAEIRASANLAATKMSECVLGQSKRVDFCGKGYHLMSFPQ<br>SAPHGVVFLHVTYVPAQEKNFTTAPAICHDGKAHFPREGVFVSNGTHWFVTQRNFY<br>EPQIITTDNTFVSGNCDVVIGIVNNTVYDPLQPELDSFKEELDKYFKNHTSPDVDLGDI<br>SGINASVVNIQKEIDRLNEVAKNLNESLIDLQELGKYEQYIKWPWYIWLGFIAGLIAIV<br>MVTIMLCCMTSCCSCLKGCCSCGSCCKFDEDDSEPVLKGVKLHYT	S1 subunit<br><br>N-Terminus<br><br>Receptor Binding Domanin<br><br>Receptor Binding Motif<br><br>S2 Subunit<br><br>Fusion Peptide<br><br>Heptad-Repeat1<br><br>Heptad-Repeat 2

1.2 Supplementary Figures

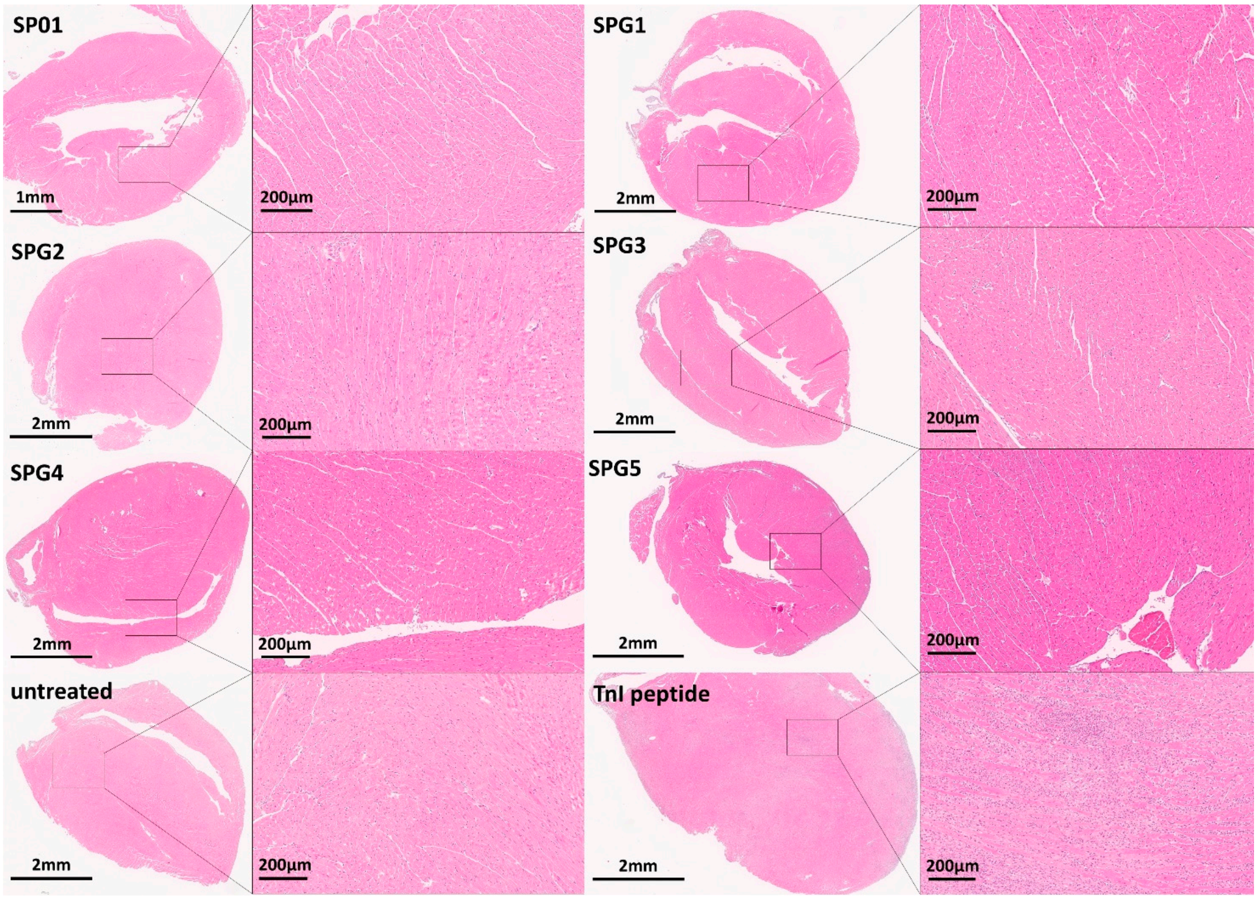

Figure S1: Representative images of single and pooled SARS-CoV-2 spike protein peptide immunized mouse hearts in 2- and 20-fold magnification.

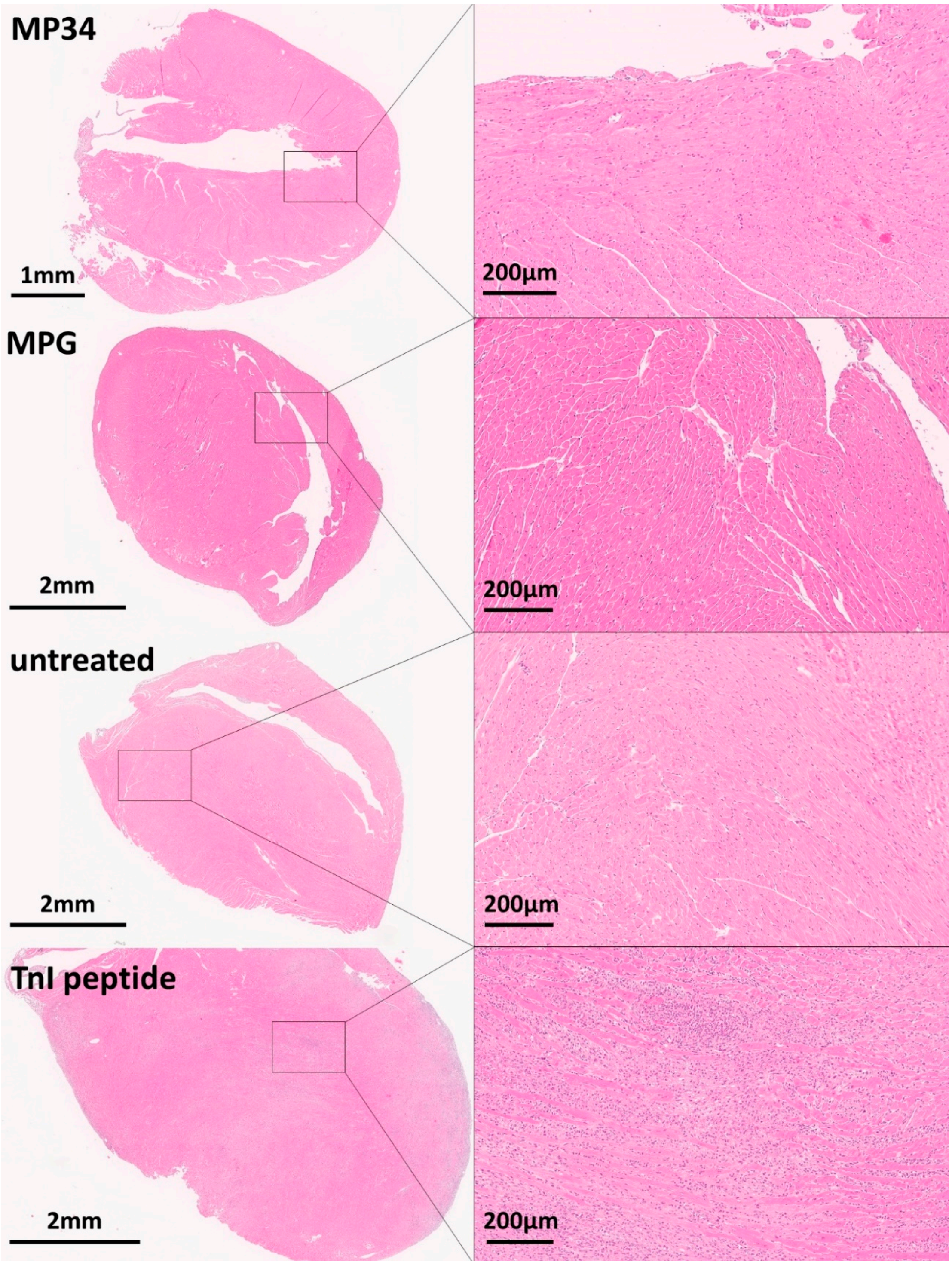

Figure S2: Representative images of single and pooled SARS-CoV-2 membrane protein peptide immunized mouse hearts in 2- and 20-fold magnification.

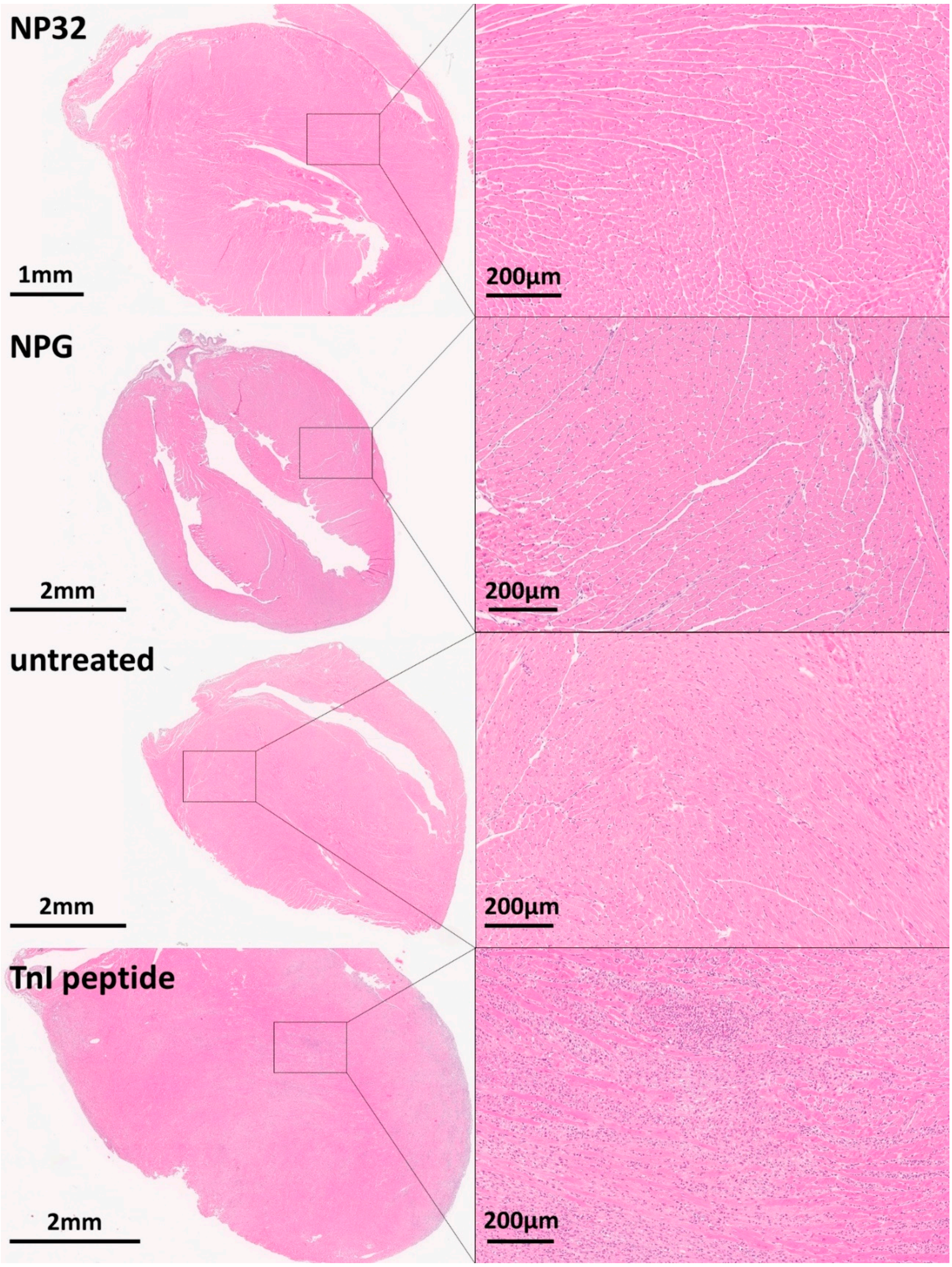

Figure S3: Representative images of single and pooled SARS-CoV-2 nucleocapsid protein peptide immunized mouse hearts in 2- and 20-fold magnification.

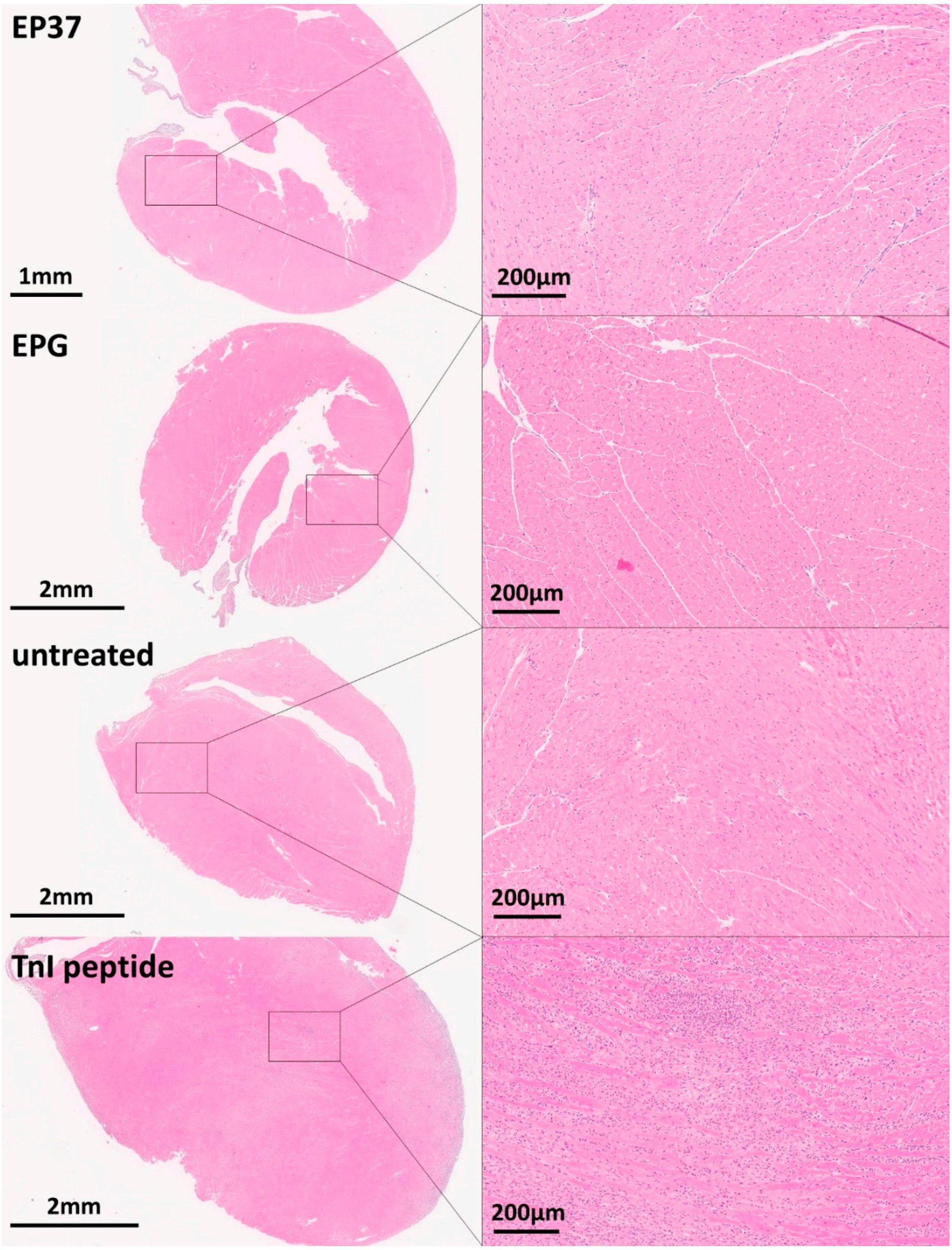

Figure S4: Representative images of single and pooled SARS-CoV-2 envelope protein peptide immunized mouse hearts in 2- and 20-fold magnification.

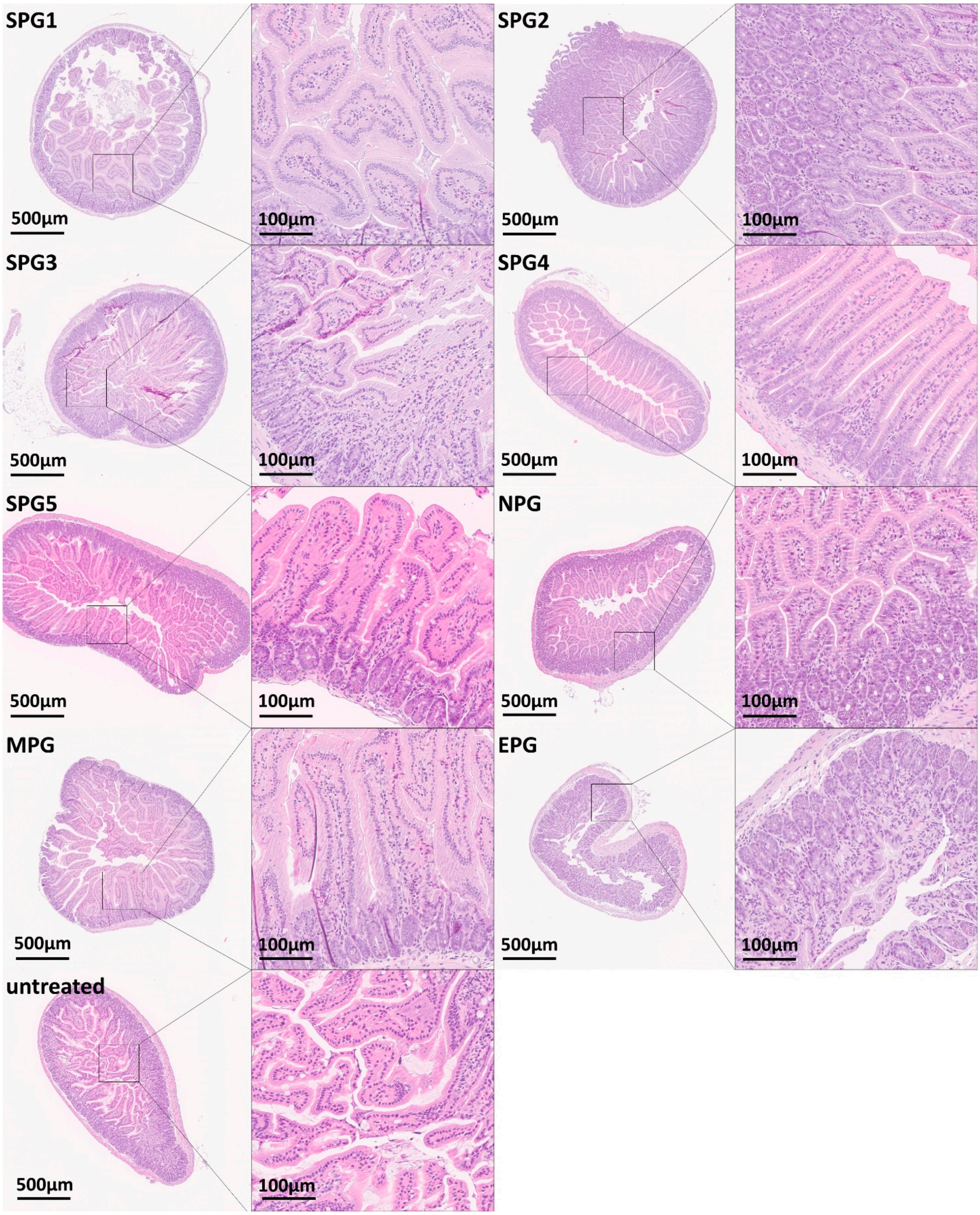

Figure S5: Histopathological examination of the intestine of the animals treated with the pooled peptide sequences SPG, MPG, NPG and EPG of SARS-CoV-2 as well as untreated animals in 4x and 20x magnification. Paraffin-embedded intestine was cut in 3 µm sections and stained with HE.

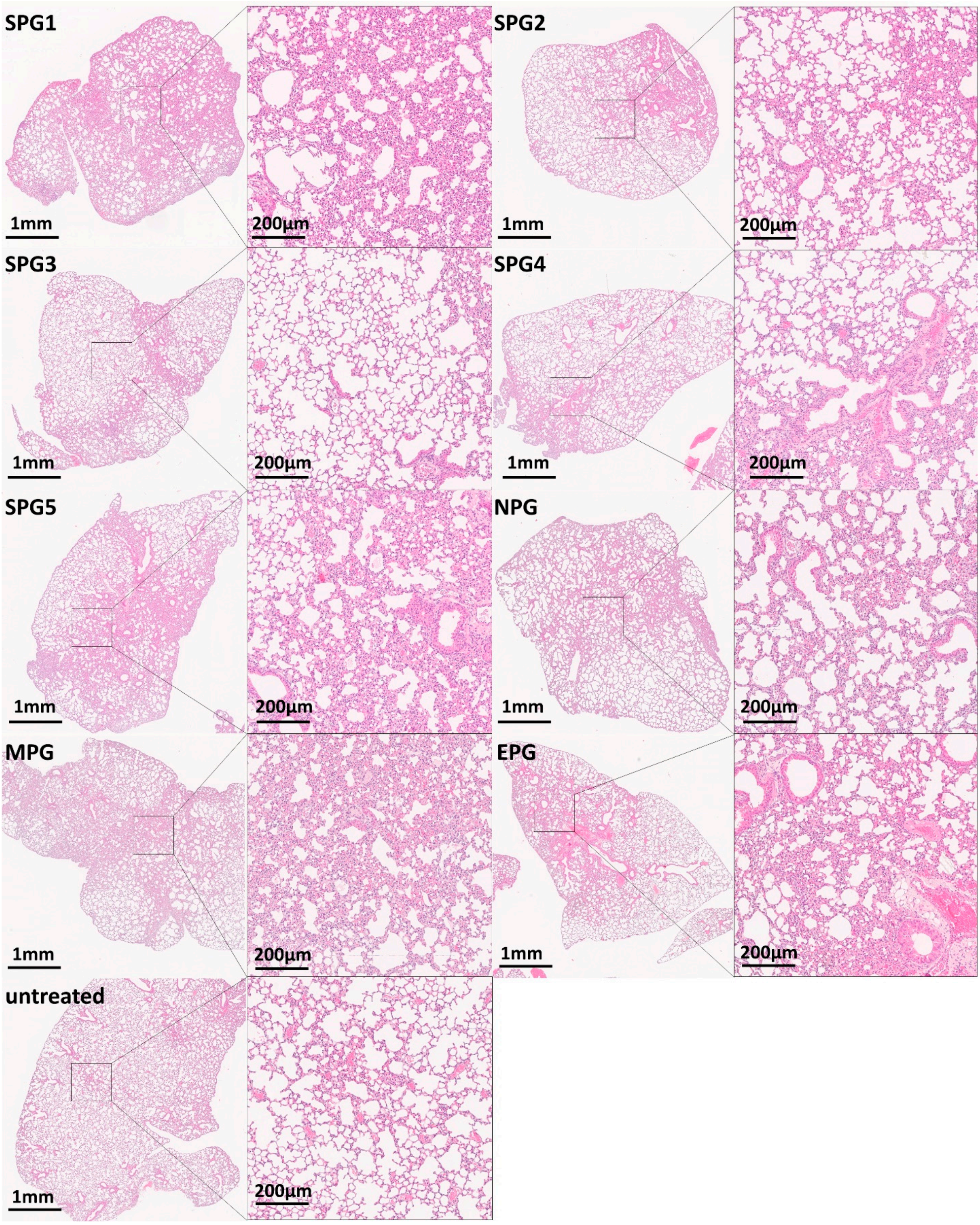

Figure S6: Histopathological examination of the lung of the animals treated with the pooled peptide sequences SPG, MPG, NPG and EPG of SARS-CoV-2 as well as untreated animals in 4x and 20x magnification. Paraffin-embedded intestine was cut in 3 µm sections and stained with HE.

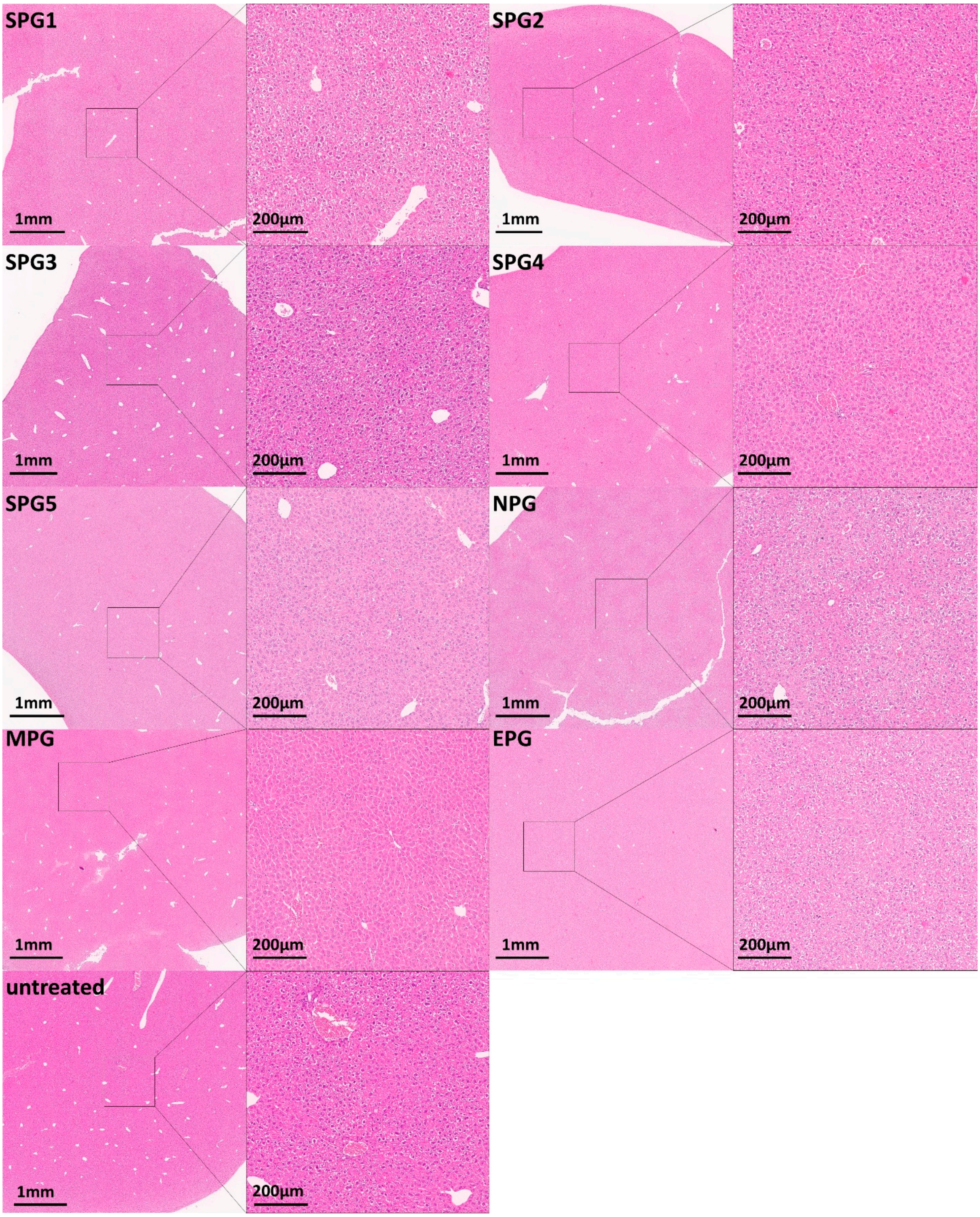

Figure S7: Histopathological examination of the liver of the animals treated with the pooled peptide sequences SPG, MPG, NPG and EPG of SARS-CoV-2 as well as untreated animals in 4x and 20x magnification. Paraffin-embedded intestine was cut in 3 µm sections and stained with HE.

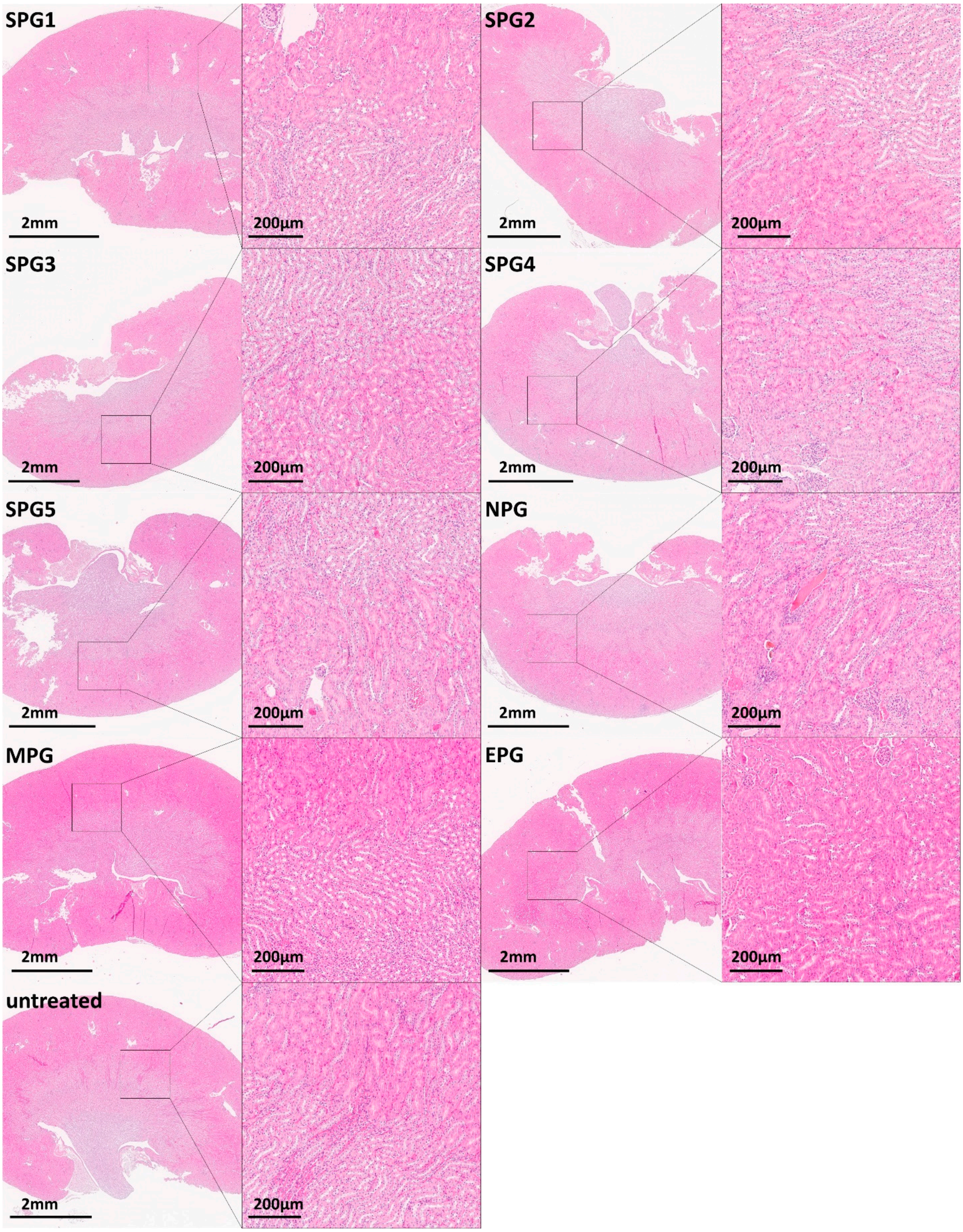

Figure S8: Histopathological examination of the kidney of the animals treated with the pooled peptide sequences SPG, MPG, NPG and EPG of SARS-CoV-2 as well as untreated animals in 4x and 20x magnification. Paraffin-embedded intestine was cut in 3 µm sections and stained with HE.

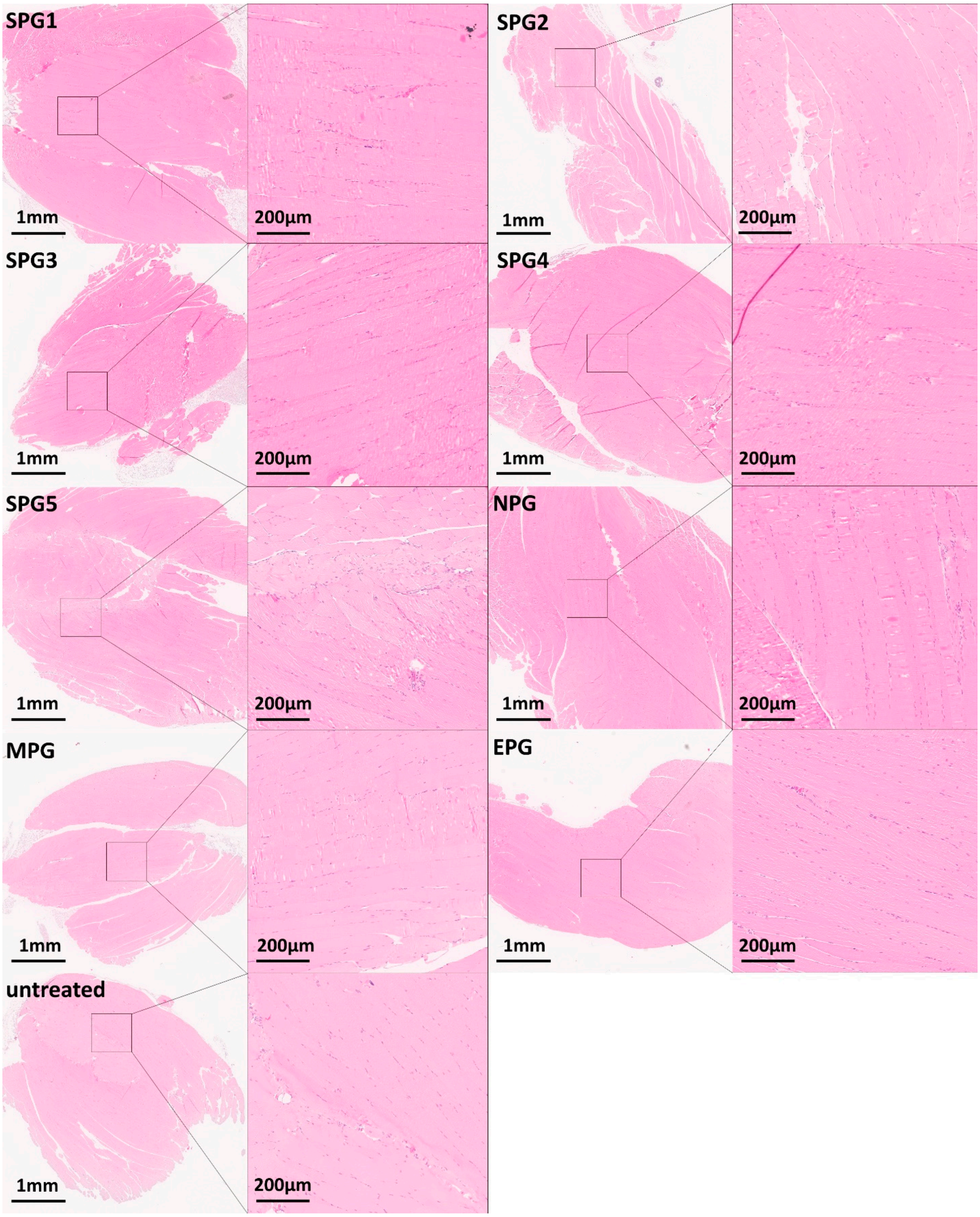

Figure S9: Histopathological examination of the muscle of the animals treated with the pooled peptide sequences SPG, MPG, NPG and EPG of SARS-CoV-2 as well as untreated animals in 4x and 20x magnification. Paraffin-embedded intestine was cut in 3 µm sections and stained with HE.

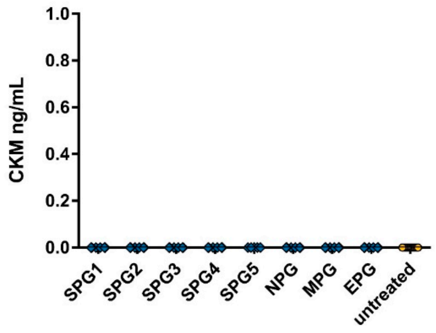

Figure S10: Evaluation of creatine kinase M (CKM) in pooled SPG1-5, NPG, MPG, EPG and untreated animals.

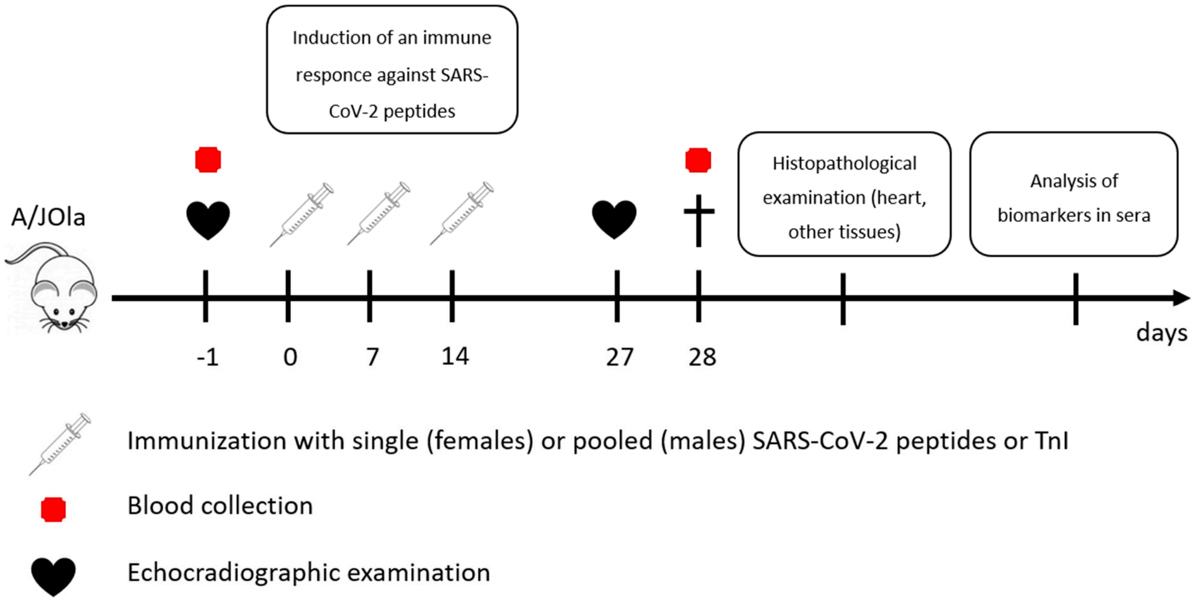

Figure S11: Schematic overview of the immunization with 150 µg single or pooled SARS-CoV-2 peptides in A/Jola mice.
